# Pharmacokinetics of Ganaplacide and Lumefantrine in Adults, Adolescents, and Children with *Plasmodium falciparum* Malaria Treated with Ganaplacide Plus Lumefantrine Solid Dispersion Formulation: Analysis of Data from a Multinational Phase 2 Study

**DOI:** 10.1002/jcph.6138

**Published:** 2024-09-29

**Authors:** Ramachandra Sangana, Bernhards Ogutu, Adoke Yeka, Sylvia Kusemererwa, Halidou Tinto, Andre Offianan Toure, Afizi Kibuuka, Moussa Lingani, Carlos Lourenço, Ghyslain Mombo‐Ngoma, Videlis Nduba, Tiacoh Landry N'Guessan, Guétawendé Job Wilfried Nassa, Mary Nyantaro, Lucas Otieno Tina, Anup Anvikar, Abhinav Sinha, Grace Kaguthi, Bakary Fofana, Martin Peter Grobusch, Myriam El Gaaloul, Anne Claire Marrast, Rashidkhan Pathan, Havana Chikoto, Katalin Csermak, Celine Risterucci, Guoqin Su, Cornelis Winnips, Jie Zhang, Julia Zack

**Affiliations:** ^1^ PK Sciences, Biomedical Research, Novartis Cambridge MA USA; ^2^ Centre for Clinical Research Kenya Medical Research Institute Kisumu, Kenya and CREATES, Strathmore University Nairobi Kenya; ^3^ Infectious Diseases Research Collaboration Kampala Uganda; ^4^ Medical Research Council/Uganda Virus Resea rch Institute and London School of Hygiene and Tropical Medicine Uganda Research Unit Entebbe Uganda; ^5^ Institut de Recherche en Science de la Santé ‐ Unité de Recherche Clinique de Nanoro (IRSS‐URCN) Nanoro Burkina Faso; ^6^ Department of Parasitology‐Mycology Institut Pasteur de Côte d'Ivoire Abidjan Côte d'Ivoire; ^7^ Infectious Diseases Research Collaboration (IDRC) Kampala Uganda; ^8^ Chókwè Health Research and Training Center/Centro de Investigação e Treino em Saúde de Chókwè (CITSC) National Institute of Health Mozambique; ^9^ Centre de Recherches Médicales de Lambaréné (CERMEL) Lambaréné Gabon; ^10^ Department of Implementation Research Bernhard Nocht Institute for Tropical Medicine and Department of Medicine University Medical Centre Hamburg‐Eppendorf Hamburg Germany; ^11^ Kenya Medical Research Institute‐Centre for Respiratory Diseases Research (KEMRI‐CRDR) Nairobi Kenya; ^12^ Centre for Clinical Research Kenya Medical Research Institute/US Army Medical Research Directorate Kisumu Kenya; ^13^ ICMR‐National Institute of Malaria Research New Delhi India; ^14^ Malaria Research and Training Center Bamako Mali; ^15^ Department of Infectious Diseases Center of Tropical Medicine and Travel Medicine, Amsterdam University Medical Centers, University of Amsterdam Amsterdam The Netherlands; ^16^ Institute of Tropical Medicine University of Tubingen Tubingen Germany; ^17^ Medicines for Malaria Venture Geneva Switzerland; ^18^ Novartis Healthcare Pvt. Ltd. Hyderabad India; ^19^ Novartis Pharmaceuticals Corporation East Hanover NJ USA; ^20^ Novartis Pharma AG Basel Switzerland; ^21^ PK Sciences, Biomedical Research Novartis East Hanover NJ USA

**Keywords:** ganaplacide, lumefantrine, pharmacokinetics, *Plasmodium falciparum* malaria

## Abstract

The novel antimalarial ganaplacide combined with lumefantrine solid dispersion formulation (LUM‐SDF) was effective and well tolerated in the treatment of uncomplicated falciparum malaria in adults, adolescents, and children in a multinational, prospective, randomized, active‐controlled Phase II study conducted between August 2017 and June 2021 (EudraCT 2020‐003284‐25, Clinicaltrials.gov NCT03167242). Pharmacokinetic data from that study are reported here.

The trial comprised three parts: a run‐in part in 12 adult/adolescent patients treated with a single dose of ganaplacide 200 mg plus LUM‐SDF 960 mg assessed potential pharmacokinetic (PK) interactions between ganaplacide and lumefantrine; in Part A, adult/adolescent patients received one of the six ganaplacide‐LUM‐SDF regimens or artemether‐lumefantrine; and in Part B, three dose regimens identified in Part A, and artemether‐lumefantrine, were assessed in children aged 2 to <12 years, with body weight ≥10 kg. A rich blood sampling schedule was used for all 12 patients in the PK run‐in part and a subset of patients (N = 32) in Part A, with sparse sampling for remaining patients in Parts A (N = 275) and B (N = 159). Drug concentrations were determined by a validated protein precipitation and reverse phase liquid chromatography with tandem mass spectrometry detection method. Parameters including AUC_inf_, AUC_last_, AUC_0‐t_, *C*_max,_ and t_max_ were reported where possible, using non‐compartmental analysis.

In the PK run‐in part, there was no notable increase in ganaplacide or lumefantrine exposure when co‐administered. In Parts A and B, ganaplacide exposures increased with dose, but lumefantrine exposure was numerically under dose‐proportional. Lumefantrine exposure was higher with ganaplacide‐LUM‐SDF than with artemether‐lumefantrine, although high variability was observed. Ganaplacide and lumefantrine exposures (*C*_max_ and AUC_0‐24 h_) were comparable across age and body weight groups. Drug exposures needed for efficacy were achieved using the dose regimen 400 mg ganaplacide plus lumefantrine 960 mg once daily for 3 days under fasted conditions.

## Introduction

While global malaria control efforts have made significant advances in reducing the impact of the disease, progress may have stalled in recent years. The global malaria case incidence declined from 2000 to 2019, but increased in 2020 and remained stable until 2022. Similarly, the malaria mortality rate halved from about 29 deaths per 100,000 population at risk in 2000 to 14 in 2019. In 2020, the mortality rate increased to 15.2, before decreasing slightly to 14.3 in 2022.^1^ Antimalarial drugs have played an important role in malaria control, and artemisinin‐based combination therapies (ACTs) have been the mainstay of treatment for several years. The recent emergence and spread of partial resistance to artemisinins, however, threaten the utility of ACTs.^1^ As a result, there is an urgent need for new antimalarials with novel mechanisms of action.

Here we report pharmacokinetic data from a recent prospective, international, randomized, active‐controlled study (EudraCT 2020‐003284‐25, Clinicaltrials.gov NCT03167242). This study showed that a combination of the novel imidazolopiperazine antimalarial ganaplacide with lumefantrine solid dispersion formulation (LUM‐SDF) was effective and well tolerated in the treatment of uncomplicated *Plasmodium falciparum* malaria in adults, adolescents, and children, with similar efficacy and safety to artemether‐lumefantrine.^2^ This study also identified treatment regimens to be tested in a confirmatory Phase III trial (ongoing study NCT05842954).

Ganaplacide (KAF156) has in vitro^3^, ^4^ and in vivo^5^, ^6^ activity against *P. falciparum* and *P. vivax*. Ganaplacide shows activity against asexual blood stages, hepatic and sexual stages of *Plasmodium*, with transmission‐blocking activity observed in vitro^7^ and in rodent models.^3^ Ganaplacide also demonstrated prophylactic efficacy in a controlled human malaria infection (CHMI) model.^8^ In previous studies in humans with ganaplacide monotherapy, time to maximum serum concentration (t_max_) was 0.9 to 2.8 h in a CHMI study^8^ and approximately 3 h in a study in malaria patients,^5^ with terminal half‐lives (t_1/2_) of 37 to 69 and approximately 44 h, respectively. In healthy volunteers, food intake did not affect the extent of ganaplacide absorption, but geometric mean C_max_ was reduced by approximately 20% and median T_max_ delayed from 3.0 to 6.0 h under fed conditions.^9^

Combinations of antimalarials are recommended to reduce the risk of development of resistance.^10^ One of the current mainstays of treatment is the ACT artemether‐lumefantrine.^10^ Artemether‐lumefantrine is a WHO‐approved first‐line treatment for uncomplicated *P. falciparum* malaria in adults, including breastfeeding women and pregnant women (in second and third trimesters) and children, including infants. The conventional formulation of lumefantrine used in this combination, however, requires twice‐daily dosing, which may impair adherence to treatment. A new solid dispersion form of lumefantrine (LUM‐SDF) has been developed; in a study in healthy adult volunteers this form increased the bioavailability of lumefantrine and provided an improved absorption profile compared to the conventional formulation used in artemether‐lumefantrine. While LUM‐SDF showed a positive food effect as observed with the conventional formulation, the bioavailability of LUM‐SDF under fasted conditions was comparable with that of the conventional formulation under fed conditions. In addition, LUM‐SDF permits once‐daily dosing while providing similar exposure to the conventional formulation.^11^

## Methods

The study protocol and amendments were reviewed and approved by ethics committees and health authorities at each center, and all patients or their parents or guardians gave written informed consent. The study design and patient population have been described previously.^2^ This was a multicenter, randomized, parallel‐group, open‐label trial. The trial was performed in three parts. In each part of the study, ganaplacide/LUM‐SDF was administered under fasting conditions (no food intake for 3 h before and 4 h after dosing) and artemether‐lumefantrine was administered under fed conditions as per the prescribing information.

The initial PK run‐in part was performed in 12 adult/adolescent patients. This part of the study was conducted to assess potential PK interaction between ganaplacide and lumefantrine when co‐administered, specifically any potential for increase in ganaplacide exposure. An increase <1.4‐fold in AUC_0‐24 h_ was considered not to be of clinical concern and would not warrant a dose adjustment for Part A as the highest single and multiple doses planned in Part A of the study were 800 and 400 mg, respectively; these represent 2/3 of the highest previously tested 1200 mg single dose and 600 mg multiple dose, which had acceptable safety profiles. A geometric mean AUC_0‐24 h_ reference value of 4.930 µg•h/mL was used as the basis for this calculation, based on a geometric mean AUC_0‐24 h_ 9.860 µg•h/mL observed after administration of 400 mg ganaplacide in patients with *P. falciparum* malaria in a previous study (CKAF156X2201). This historical value from Study CKAF156X2201 was chosen as the current study did not include a monotherapy arm for comparison, and the pharmacokinetics of ganaplacide as monotherapy have previously been characterized and shown to be reproducible across studies in both healthy volunteers and malaria patients.^12^, ^13^, ^14^ Using a historical reference value for comparison should therefore not impact the outcome of the current study.

In this part of the study, patients received single doses of ganaplacide 200 mg and LUM‐SDF 960 mg. A sample size of 12 patients was used as this would provide 80% probability that the observed half width of two‐sided 90% CI for log AUC_0‐24 h_ was ≤0.155 or 1.17 in terms of ratio, and would also provide 80% probability that the observed half width of two‐sided 90% CI for log C_max_ was ≤0.126 or 1.13 in terms of ratio.

Part A was conducted on successful completion of the PK run‐in part. Part A aimed to identify effective and well‐tolerated treatment regimens in adult/adolescent patients. Six ganaplacide plus LUM‐SDF dose regimens were evaluated:

- Once‐daily ganaplacide 400 mg/LUM‐SDF 960 mg for 1, 2, or 3 days;
- Once‐daily ganaplacide 800 mg/LUM‐SDF 960 mg as a single dose;
- Once‐daily ganaplacide 200 mg/LUM‐SDF 480 mg for 3 days;
- Once‐daily ganaplacide 400 mg/LUM‐SDF 480 mg for 3 days.

Artemether‐lumefantrine was used as a control treatment, administered twice daily for 3 days as per standard prescribing information.

In Part B, following review of the efficacy, tolerability and safety outcomes in Part A, three dose regimens of ganaplacide/LUM‐SDF, with artemether‐lumefantrine as a control, were assessed for efficacy, tolerability, and safety in children aged 2 to <12 years with body weight ≥10 kg. The selected dose regimens were ganaplacide 400 mg/LUM‐SDF 960 mg, given once daily for 1, 2, or 3 days. Both ganaplacide 400 mg/LUM‐SDF 960 mg and artemether‐lumefantrine doses were adjusted for body weight in Part B using the same weight categories as are standard for artemether‐lumefantrine. After treatment, patients were followed up until 42 days after the first dose of study medication.

Samples size calculations for Parts A and B of the study were based on efficacy rather than pharmacokinetic analyses, and have been described previously.^2^

Blood samples for PK analysis were collected for rich PK sampling in the run‐in part and for a subset of patients in Part A, namely 6 patients in each dose group other than the ganaplacide 400 mg/LUM‐SDF 480 mg for 3 days group, where rich sampling was used in 12 patients. Sparse PK sampling was used for the remaining patients in Part A and all patients in Part B. Sampling schedules are shown in Table S1.

Pharmacokinetic analyses were conducted using the PK analysis set, which included all patients who had taken at least one dose of the study treatment during the treatment period, had evaluable PK parameter data, had at least 80% compliance to study treatment (as assessed by direct observation and drug reconciliation records), and did not receive prohibited medications which could impact the PK parameters of lumefantrine or ganaplacide. Prohibited medications are listed in Table S2.

Ganaplacide and lumefantrine concentrations in plasma samples were determined by a validated protein precipitation followed by reverse phase liquid chromatography with tandem mass spectrometry detection method. The lower limit of quantification (LLOQ) was 5.00 ng/mL and the upper limit of quantification (ULOQ) was 5000 ng/mL, using 20 µL of plasma for ganaplacide. For lumefantrine the LLOQ was 50.0 ng/mL, and ULOQ was 20,000 ng/mL, also based on 20 µL of plasma.

For both ganaplacide and lumefantrine, parameters such as AUC_inf_, AUC_last_, AUC_0‐t_, C_max_, and t_max_ were reported, where possible for the patients with rich PK data using non‐compartmental method of analysis (using Phoenix 6.4 or higher). Two‐sided 90% CIs for AUC_0‐24 h_, C_max_, and t_max_ were provided. PK parameters of ganaplacide and lumefantrine were calculated by part and treatment cohort. Non‐compartmental PK analysis for patients with sparse data was also conducted and feasible PK parameters were reported.

## Results

The study was conducted between August 2, 2017 (first patient recruited) and June 8, 2021 (last patient completed the study). Patients were recruited at 13 study centers in 10 countries: Uganda (2 centers), Kenya (2 centers), Cote d'Ivoire (2 centers), Vietnam, Thailand, Burkina Faso, Gabon, Mozambique, Mali, and India (1 center each). A total of 524 patients participated: 12 patients in the PK run‐in part (all recruited in Mali), 337 patients in Part A, and 175 in Part B. All patients in the PK run‐in part completed the study, as did 334/337 (99.1%) in Part A and 162/175 (92.6%) in Part B. All 12 patients in the PK run‐in part were included in the PK analysis set, as were 307/337 (91.1%) of patients in Part A (32 patients with rich PK sampling and 275 with sparse sampling), and 159/175 (90.9%) in Part B (all sparse sampling). Demographic and baseline characteristics of the study population have been reported previously.^2^

Table 1 summarizes PK parameters for ganaplacide and lumefantrine in the PK run‐in part. Geometric mean ganaplacide *C*_max_ was 412 ng/mL (90% CI 338, 502), t_max_ was 3.98 h, and t_1/2_ was 23.7 h. Corresponding values for lumefantrine were 7570 ng/mL (90% CI 4540, 12,600), 7.65 h, and 34.2 h, respectively. The geometric mean ganaplacide AUC_0‐24h_ was 5.35 µg h/mL; comparing this with the historical reference value of 4.930 µg•h/mL resulted in a relative exposure factor of 1.09, indicating no increase in ganaplacide exposure in the presence of lumefantrine. The relative exposure factor was below the prospectively defined threshold for clinical relevance (exposure factor of ≥1.4), therefore no dose adjustment of ganaplacide was considered necessary for Part A.

**Table 1:** Summary Statistics for PK Parameters by Sampling Type, Analyte, and Treatment Group (PK Run‐In Part; PK Analysis Set)

Parameter	n	Mean (SD)	CV% mean	Geometric mean (90% CI)^a^	CV% Geometric mean	Median	Min	Max
Ganaplacide								
Day 1 AUC_0‐24 h_ (µg•h/mL)	11	5.6 (2)	35.5	5.4 (4.5, 6.4)	34.8	4.8	3.6	9.7
C_max_ (ng/mL)	11	436 (151)	34.5	412 (338, 502)	37.5	456	244	676
t_max_ (h)	12	4.2 (1.6)	36.8	4.0	37.4	3.0	2.9	6.1
AUC_inf_ (µg•h/mL)	10	10.5 (4.9)	46.4	9.6 (7.6, 12.2)	43.2	9.3	5.8	19.8
t_1/2_ (h)	11	25 (8.8)	35.2	23.7	35.6	22.6	12.6	41.3
Total Cl/F observed (mL/h)	10	22,300 (8060)	36.2	20,800	43.2	21,600	10,100	34,500
Lumefantrine								
Day 1 AUC_0‐24 h_ (µg•h/mL)	12	146 (108)	73.7	106 (62.8, 179)	133.1	114	7.2	417
C_max_ (ng/mL)	12	10,100 (6490)	63.9	7570 (4540, 12,600)	128.4	8920	539	24,700
t_max_ (h)	12	8.1 (2.9)	35.4	7.7	33.7	6.2	6.0	12
AUC_inf_ (µg•h/mL)	12	279 (241)	86.3	192 (111, 331)	141.6	191	13.2	906
t_1/2_ (h)	12	36.5 (11.6)	31.6	34.2	43.6	38.3	14.3	49.5
Total Cl/F observed (mL/h)	12	10,300 (19,700)	191.6	5000	141.6	5030	1060	72,500

n, number of patients with non‐missing values.

CV% = coefficient of variation (%) = SD/mean × 100; CV% geo mean = (sqrt (exp (variance for log transformed data) − 1)) × 100.

90% CI for the geometric mean is calculated based on the log transformation of the reduction ratios assuming log normal distribution and transformed back to the exponential.

^a^ 90% CI was calculated for C_max_, AUC_0‐24 h_, and AUC_inf_.

Lumefantrine exposure in the PK run‐in part (mean AUC_0‐24 h_ 146 µg•h/mL, range 7.19 to 417 µg•h/mL) was numerically higher (approximately 1.4‐fold) than previously observed in healthy individuals (mean AUC_0‐24 h_ 107 µg•h/mL at a lumefantrine dose of 960 mg).^9^ This difference was not considered to be clinically significant given the small sample size in both studies, the known variability of lumefantrine pharmacokinetics, and the potential difference between healthy individuals and malaria patients.^11^, ^12^ Lumefantrine doses in Part A were therefore not adjusted.

Table 2 presents PK parameters for ganaplacide and lumefantrine in Part A, based on both rich and sparse sampling. In general, AUC_0‐24 h_ from sparse sampling was comparable to rich sampling for both ganaplacide and lumefantrine. Ganaplacide exposures increased with dose, but lumefantrine exposures appeared to be under dose‐proportional, as is also illustrated in Figure S1. High variability in exposure was observed, especially for lumefantrine.

**Table 2:** Summary Statistics for PK Parameters by Analyte, Sampling Type, and Treatment Group (Part A; PK Analysis Set)

		Geo Mean (CV% Geo Mean) [n]							
			C_max_ (ng/mL)		D1 AUC_0‐24 h_ (µg•h/mL)		D2/D3 AUC_0‐24 h_ (µg•h/mL)^c^		Cl/F Observed (mL/h)
Treatment Regimen	C_168 h_ (ng/mL) Arithmetic Mean (SD) [n]^a^, ^b^	C_168 h_ (ng/mL)^b^	Rich	Sparse	Rich	Sparse	Rich	Sparse	Rich
Ganaplacide									
Gan 400 mg/LUM 960 mg, 1 day	11.6 (10.1) [39]	9.0 (95.6) [39]	237 (850.5) [5]	653 (43.9) [39]	2.2 (10,331) [5]	9.8 (41.5) [38]	NA	NA	22,100 (89.1) [4]
Gan 800 mg/LUM 960 mg, 1 day	26.4 (17.8) [44]	21.4 (74.7) [44]	1100 (54.2) [6]	1470 (46.5) [44]	18.3 (64.8) [6]	21.7 (41.7) [44]	NA	NA	18,500 (67.4) [6]
Gan 400 mg/LUM 960 mg, 2 days	31 (21.8) [40]	25.6 (68.6) [40]	1150 (16.7) [4]	1060 (83.9) [40]	10.7 (21.2) [4]	9.95 (131.9) [41]	16.4 (18.6) [4]	17.3 (117.2) [39]	NA
Gan 200 mg/LUM 480 mg, 3 days	28.9 (17.1) [41]	24.9 (59.8) [41]	782 (13) [2]	665 (30.3) [43]	6.31 (16.7) [2]	5.91 (29.2) [43]	11.9 (9.4) [2]	10.8 (31.7) [42]	NA
Gan 400 mg/LUM 480 mg, 3 days	78.4 (66.9) [39]	59.1 (85.7) [39]	1170 (40.3) [9]	1470 (30.9) [36]	9.3 (29.7) [8]	11 (79.3) [36]	19.3 (40.3) [9]	24.7 (33.2) [35]	NA
Gan 400 mg/LUM 960 mg, 3 days	68.9 (46.3) [40]	55.2 (77.5) [40]	1180 (26.8) [2]	1320 (32.7) [41]	10.4 (46.6) [2]	10.9 (57.4) [41]	19.1 (0.9) [2]	22.2 (32) [39]	NA
Lumefantrine									
Gan 400 mg/LUM 960 mg, 1 day	220 (177) [39]	161 (111.1) [39]	6750 (146) [5]	4820 (88.9) [39]	96.8 (147.7) [5]	73.7 (91.1) [39]	NA	NA	4520 (143.2) [5]
Gan 800 mg/LUM 960 mg, 1 day	229 (179) [45]	168 (106.9) [45]	4330 (177.5) [6]	4350 (134.2) [45]	65.7 (192) [6]	70.5 (133.2) [45]	NA	NA	6000 (229.6) [6]
Gan 400 mg/LUM 960 mg, 2 days	698 (381) [39]	588 (71.1) [39]	17,200 (69.3) [4]	12,500 (80.9) [38]	211 (60) [4]	88.8 (117.6) [40]	205 (81.2) [4]	194 (89) [38]	NA
Gan 200 mg/LUM 480 mg, 3 days	753 (397) [43]	655 (59.1) [43]	10,400 (119.1) [3]	12,500 (52.2) [44]	75.4 (11) [3]	72.1 (88.7) [42]	150 (151.9) [3]	194 (66.9) [43]	NA
Gan 400 mg/LUM 480 mg, 3 days	931 (668) [40]	723 (85.4) [40]	6920 (86.9) [9]	12,700 (76.2) [35]	38.1 (214.4) [8]	60.7 (103.8) [35]	117 (86.3) [9]	212 (82.6) [35]	NA
Gan 400 mg/LUM 960 mg, 3 days	1100 (819) [39]	884 (75.7) [39]	14,200 (50) [4]	14,500 (72.3) [38]	108 (97.9) [4]	95.4 (118.1) [36]	246 (34.4) [4]	230 (76.6) [37]	NA
Artemether‐lumefantrine	816 (582) [22]	695 (59.1) [22]	NA	10,400 (43.8) [24]	NA	NA	NA	NA	NA

Gan, ganaplacide; NA, either not determined or not reportable for specific regimen.

^a^ Arithmetic mean was also reported for C_168 h_ as there were a number of concentrations less than the lower limit of quantification.

^b^ C_168 h_ includes all evaluable patients (sparse and rich).

^c^ Day 2 AUC_0‐24 h_ for the 2‐day regimen and Day 3 AUC_0‐24 h_ for the 3‐day regimen.

Geometric mean half‐life of ganaplacide, based on rich PK sampling, ranged between 23.7 and 33.1 h; for lumefantrine, geometric mean half‐life ranged from 30.9 to 80.9 h. Geometric mean total clearance, also based on rich PK sampling, ranged from 18,500 to 22,100 mL/h for ganaplacide and from 4520 to 6000 mL/h for lumefantrine.

Table 3 summarizes PK parameters from Parts A and B of the study for ganaplacide‐lumefantrine regimens that were used in both parts of the study. Comparable exposures were observed for both ganaplacide and lumefantrine between the adults and adolescents in Part A and the children in Part B. For example, ganaplacide geometric mean C_max_ (CV%) with the 3‐day regimen was 1320 (32.7) ng/mL in Part A and 1380 (29.7) ng/mL in Part B; corresponding Day 3 AUC_0‐24 h_ (CV%) values were 22.2 (32) and 23.7 (32.9) µg•h/mL, respectively. *C*_168h_ for ganaplacide was lower in Part B than Part A. High variability in lumefantrine exposure was observed in both Part A and Part B; geometric mean (CV%) Day 3 AUC_0‐24 h_ values were 230 (76.6) µg•h/mL in Part A and 369 (81.4) µg•h/mL in Part B.

**Table 3:** Summary Statistics for PK Parameters by Analyte and Treatment (Part A and Part B; PK Analysis Set)

		Ganaplacide					Lumefantrine				
Treatment Regimen	Part	C_168 h_ (ng/mL) Arithmetic Mean (SD) [n]^a^	C_168 h_ (ng/mL) (CV% Geo Mean) [n]^a^	C_max_ (ng/mL) (CV% Geo Mean) [n] (Sparse)	D1 AUC_0‐24 h_ (µg•h/mL) (CV% Geo Mean) [n] (Sparse)	D2/3 AUC_0‐24 h_ (µg•h/mL) (CV% Geo Mean) [n] (Sparse)	C_168 h_ (ng/mL) Arithmetic Mean (SD) [n]	C_168 h_ (CV% Geo Mean) [n]	C_max_ (ng/mL) (CV% Geo Mean) [n] (Sparse)	D1 AUC_0‐24 h_ (µg•h/mL) (CV% Geo Mean) [n] (Sparse)	D2/3 AUC_0‐24 h_ (µg•h/mL) (CV% Geo Mean) [n] (Sparse)
Gan 400 mg/LUM 960 mg, 1 day	A	11.6 (10.1) [39]	9.0 (95.6) [39]	653 (43.9) [39]	9.8 (41.5) [38]	NA	220 (177) [39]	161 (111.1) [39]	4820 (88.9) [39]	73.7 (91.1) [39]	NA
	B	2.61 (3.92) [45]	3.6 (59.3) [45]	714 (49.4) [48]	11 (47.7) [48]	NA	324 (240) [38]	238 (108.4) [38]	7600 (155.3) [40]	120 (153.6) [40]	NA
Gan 400 mg/LUM 960 mg, 2 days	A	31 (21.8) [40]	25.6 (68.6) [40]	1060 (83.9) [40]	10.0 (131.9) [41]	17.3 (117.2) [39]	698 (381) [39]	588 (71.1) [39]	12,500 (80.9) [38]	88.8 (117.6) [40]	194 (89) [38]
	B	11 (8.2) [43]	9.0 (89.5) [43]	1060 (48.4) [46]	NA	17.5 (49.6) [46]	861 (476) [40]	732 (67.7) [40]	16,200 (71.4) [43]	NA	282 (68.7) [43]
Gan 400 mg/LUM 960 mg, 3 days	A	68.9 (46.3) [40]	55.2 (77.5) [40]	1320 (32.7) [41]	10.9 (57.4) [41]	22.2 (32) [39]	1100 (819) [39]	884 (75.7) [39]	14,500 (72.3) [38]	95.4 (118.1) [36]	230 (76.6) [37]
	B	33.2 (23.3) [39]	25.8 (91.3) [39]	1380 (29.7) [41]	NA	23.7 (32.9) [41]	1700 (1100) [36]	1320 (98) [36]	19,900 (81.8) [37]	NA	369 (81.4) [37]

Gan, ganaplacide; NA, either not determined or not reportable for specific regimen.

C_max_ and AUC_0‐24 h_ were reported as geo means.

^a^ C_168 h_ includes all evaluable patients (sparse and rich).

No differences between body weight groups in exposure, in terms of C_max_ and AUC_0‐24h_, were apparent for ganaplacide or lumefantrine, although (consistent with the differences observed between Part A and B), a decreasing trend for ganaplacide C_168 h_ was observed in lower body weight groups (Tables 4 and 5). Lumefantrine exposure, in terms of C_168 h_, was numerically higher for the 3‐day ganaplacide 400 mg/LUM‐SDF 960 mg regimen than with artemether‐lumefantrine for all body weight groups apart from the 10 to <15 kg group, where C_168 h_ values were comparable (Table 5). When analyzed by age group, no difference in exposure was noted for either ganaplacide or lumefantrine, but a decreasing trend for ganaplacide C_168 h_ was observed in lower age groups (Tables 6 and 7). For lumefantrine, C_168 h_ was numerically higher with the 3‐day ganaplacide 400 mg/LUM‐SDF 960 mg regimen than with artemether‐lumefantrine in all age groups other than ≥18 years, where values were comparable.

**Table 4:** Summary Statistics for PK Parameters by Weight Group and Treatment for Ganaplacide (Part A and Part B; PK Analysis Set)

Treatment Regimen	Body Weight Range (kg)	C_168 h_ (ng/mL) (Arithmetic Mean SD) [n]^a^	C_168 h_ (ng/mL) (CV% Geo Mean) [n]^a^	C_max_ (ng/mL) (CV% Geo Mean) [n] Sparse	D1 AUC_0‐24 h_ (µg•h/mL) (CV% Geo Mean) [n] Sparse	D2/D3 AUC_0‐24 h_ (µg•h/mL) (CV% Geo Mean) [n] Sparse
Ganaplacide	≥35	11.3 (10.0) [38]	8.7 (94.5) [38]	650 (44.4) [38]	9.8 (42) [37]	NA
400 mg/LUM 960 mg	25 to <35	3.3 (4.7) [17]	3.9 (71.7) [17]	766 (34.5) [18]	11.5 (32.5) [18]	NA
1 day	15 to <25	2.8 (3.7) [20]	3.75 (56.8) [20]	779 (52.3) [21]	12.3 (52.5) [21]	NA
	10 to <15	0.71 (2.0) [8]	2.77 (29.4) [8]	506 (55.9) [9]	7.61 (46) [9]	NA
Ganaplacide	≥35	31 (21.8) [40]	25.6 (68.6) [40]	1060 (83.9) [40]	9.95 (131.9) [41]	17.3 (117.2) [39]
400 mg/LUM 960 mg	25 to <35	12.5 (5.2) [11]	11.4 (51.6) [11]	1320 (25.9) [13]	NA	21.9 (26) [13]
2 days	15 to <25	13.9 (8.9) [21]	11.4 (83.4) [21]	1200 (35) [22]	NA	20.2 (35.6) [22]
	10 to <15	4.05 (4.77) [11]	4.43 (74.9) [11]	625 (49.8) [11]	NA	10.2 (50.6) [11]
Ganaplacide	≥35	68.9 (46.3) [40]	55.2 (77.5) [40]	1320 (32.7) [41]	10.9 (57.4) [41]	22.2 (32) [39]
400 mg/LUM 960 mg	25 to <35	32 (19.2) [16]	26.9 (67.9) [16]	1410 (34.9) [17]	NA	24.8 (39) [17]
3 days	15 to <25	40.1 (26) [18]	32.6 (78.3) [18]	1440 (25.2) [19]	NA	24.1 (28.8) [19]
	10 to <15	12.7 (11.6) [5]	9.72 (117) [5]	1120 (21.5) [5]	NA	19.1 (18.1) [5]

LUM, lumefantrine; NA, either not determined or not reportable for specific regimen.

*C*_max_ and AUC_0‐24 h_ were reported as geo means.

^a^ C_168 h_ includes all evaluable patients (sparse and rich).

**Table 5:** Summary Statistics for PK Parameters by Weight Group and Treatment for Lumefantrine (Part A and Part B; PK Analysis Set)

Treatment Regimen	Body Weight Range (kg)	C_168 h_ (ng/mL) (Arithmetic Mean SD) [n]1	C_168 h_ (ng/mL) (CV% Geo Mean) [n]^a^	C_max_ (ng/mL) (CV% Geo Mean) [n] Sparse	D1 AUC_0‐24 h_ (µg•h/mL) (CV% Geo Mean) [n] Sparse	D2/D3 AUC_0‐24 h_ (µg•h/mL) (CV% Geo Mean) [n] Sparse
Ganaplacide	≥35	224 (178) [38]	163 (112.6) [38]	4980 (86.5) [38]	75.8 (89.3) [38]	NA
400 mg/LUM 960 mg	25 to <35	365 (268) [14]	270 (104.1) [14]	6930 (140.7) [15]	106 (148.8) [15]	NA
1 day	15 to <25	323 (243) [18]	229 (132.6) [18]	8480 (214.4) [18]	134 (205) [18]	NA
	10 to <15	229 (151) [6]	199 (59.4) [6]	7010 (79) [7]	117 (70.9) [7]	NA
Ganaplacide	≥35	698 (381) [39]	588 (71.1) [39]	12,500 (80.9) [38]	88.8 (117.6) [40]	194 (89) [38]
400 mg/LUM 960 mg	25 to <35	852 (355) [10]	784 (45.8) [10]	20,800 (50.3) [12]	NA	352 (45) [12]
2 days	15 to <25	977 (557) [19]	836 (63.2) [19]	18,300 (64.6) [20]	NA	317 (63.9) [20]
	10 to <15	667 (379) [11]	548 (87.1) [11]	9810 (74.2) [11]	NA	178 (75.6) [11]
Ganaplacide	≥35	1100 (819) [39]	884 (75.7) [39]	14,500 (72.3) [38]	95.4 (118.1) [36]	230 (76.6) [37]
400 mg/LUM 960 mg	25 to <35	1360 (674) [14]	1150 (76.2) [14]	21,200 (41.2) [15]	NA	396 (40.3) [15]
3 days	15 to < 25	2210 (1300) [17]	1680 (124.1) [17]	20,500 (128.5) [17]	NA	371 (128.1) [17]
	10 to <15	923 (295) [5]	876 (40) [5]	15,000 (22.2) [5]	NA	292 (28.7) [5]
Artemether‐	≥35	816 (582) [22]	695 (59.1) [22]	10,400 (43.8) [24]	NA	NA
lumefantrine	25 to <35	697 (78.4) [4]	693 (12) [4]	8130 (55.9) [4]	NA	NA
	15 to <25	538 (386) [16]	441 (73.6) [16]	7500 (81) [17]	NA	NA
	10 to <15	1080 (853) [3]	763 (169.6) [3]	8180 (119.6) [3]	NA	NA

LUM, lumefantrine; NA, either not determined or not reportable for specific regimen.

C_max_ and AUC_0‐24 h_ were reported as geo means.

^a^ C_168 h_ includes all evaluable patients (sparse and rich).

**Table 6:** Summary Statistics for PK Parameters by Age Group and Treatment for Ganaplacide (Part A and Part B; PK Analysis Set)

Treatment Regimen	Age Group (years)	C_168 h_ (ng/mL) (Arithmetic Mean SD) [n]^a^	C_168 h_ (ng/mL) (CV% Geo Mean) [n]^a^	C_max_ (ng/mL) (CV% Geo Mean) [n] Sparse	D1 AUC_0‐24 h_ (µg ·h/mL) (CV% Geo Mean) [n] Sparse	D2/D3 AUC_0‐24 h_ (µg•h/mL) (CV% Geo Mean) [n] Sparse
Ganaplacide	≥18	16.3 (10.8) [20]	13.4 (78.2) [20]	648 (32) [22]	10.0 (28.3) [22]	NA
400 mg/LUM 960 mg	12 to <18	6.6 (6.7) [18]	5.8 (83.1) [18]	660 (58) [17]	9.7 (57.2) [16]	NA
1 day	6 to <12	3.4 (4.3) [28]	4.1 (64.9) [28]	743 (43.1) [29]	11.6 (41.4) [29]	NA
	2 to <6	1.6 (3.2) [18]	3.2 (48.5) [18]	672 (58.9) [19]	10.1 (56.5) [19]	NA
Ganaplacide	≥18	39.4 (27.2) [19]	32.8 (67.6) [19]	1100 (28.9) [17]	10.9 (30.1) [18]	18.4 (28) [17]
400 mg/LUM 960 mg	12 to <18	23.4 (11.5) [21]	20.5 (59.5) [21]	1030 (119.2) [23]	9.3 (218.5) [23]	16.6 (186.7) [22]
2 days	6 to <12	12.9 (7.3) [24]	11 (74.3) [24]	1260 (24.5) [27]	NA	20.5 (27.7) [27]
	2 to <6	8.7 (8.8) [19]	6.9 (99) [19]	816 (61.8) [19]	NA	14 (65.2) [19]
Ganaplacide	≥18	86.3 (45.4) [17]	73.6 (69.6) [17]	1280 (34.9) [18]	10.8 (36.6) [18]	21.5 (33) [17]
400 mg/LUM 960 mg	12 to <18	56.1 (43.6) [23]	44.6 (73.9) [23]	1360 (31.5) [23]	11 (72.2) [23]	22.8 (31.7) [22]
3 days	6 to <12	37 (19.7) [26]	31.9 (62.7) [26]	1430 (32.7) [28]	NA	24.8 (36.2) [28]
	2 to < 6	25.7 (28.4) [13]	16.9 (124.3) [13]	1290 (21.4) [13]	NA	21.5 (22.8) [13]

LUM, lumefantrine; NA, either not determined or not reportable for specific regimen.

C_max_ and AUC_0‐24 h_ were reported as geo means.

^a^ C_168 h_ includes all evaluable patients (sparse and rich).

**Table 7:** Summary Statistics for PK Parameters by Age Group and Treatment for Lumefantrine (Part A and Part B; PK Analysis Set)

Treatment Regimen	Age Group (years)	C_168 h_ (ng/mL) (Arithmetic Mean SD) [n]^a^	C_168 h_ (ng/mL) (CV% Geo Mean) [n]^a^	C_max_ (ng/mL) (CV% Geo Mean) [n] Sparse	D1 AUC_0‐24 h_ (µg•h/mL) (CV% Geo Mean) [n] Sparse	D2/D3 AUC_0‐24 h_ (µg•h/mL) (CV% Geo Mean) [n] Sparse
Ganaplacide	≥18	280 (210) [19]	210 (105.4) [19]	5890 (75.4) [21]	90 (80.4) [21]	NA
400 mg/LUM 960 mg	12 to <18	159 (119) [19]	121 (108.3) [19]	3900 (100.2) [17]	58.9 (100.1) [17]	NA
1 day	6 to <12	365 (269) [24]	263 (115.4) [24]	8140 (126.4) [25]	124 (130.3) [25]	NA
	2 to <6	254 (159) [15]	204 (92.5) [15]	6380 (211.6) [16]	107 (199.2) [16]	NA
Ganaplacide	≥18	720 (386) [19]	613 (69.7) [19]	14,700 (53.8) [17]	101 (108.5) [18]	225 (49.9) [17]
400 mg/LUM 960 mg	12 to <18	677 (385) [20]	565 (74.3) [20]	11,000 (99.5) [21]	80.1 (126.8) [22]	173 (117.5) [21]
2 days	6 to <12	878 (429) [23]	783 (52.7) [23]	19,400 (55.6) [26]	NA	331 (53) [26]
	2 to <6	837 (546) [17]	670 (87.5) [17]	12,200 (82.9) [17]	NA	220 (82.7) [17]
Ganaplacide	≥18	1030 (617) [18]	849 (75.4) [18]	12,000 (77.7) [17]	87.3 (99.6) [17]	198 (79.9) [17]
400 mg/LUM 960 mg	12 to <18	1160 (971) [21]	914 (78.1) [21]	16,900 (64) [21]	103 (138.6) [19]	260 (72.6) [20]
3 days	6 to <12	1800 (1020) [24]	1480 (81) [24]	23,000 (57) [25]	NA	427 (54.8) [25]
	2 to <6	1500 (1260) [12]	1060 (131.6) [12]	14,700 (122.4) [12]	NA	272 (125) [12]
Artemether‐	≥18	1110 (746) [10]	960 (56.3) [10]	11,400 (40.4) [12]	NA	NA
lumefantrine	12 to <18	533 (187) [11]	502 (39.9) [11]	9370 (48.7) [11]	NA	NA
	6 to <12	603 (393) [15]	500 (74.8) [15]	6950 (84.6) [15]	NA	NA
	2 to <6	731 (541) [9]	574 (87.4) [9]	9390 (60.8) [10]	NA	NA

LUM, lumefantrine; NA, either not determined or not reportable for specific regimen.

C_max_ and AUC_0‐24 h_ were reported as geo means.

^a^ C_168 h_ includes all evaluable patients (sparse and rich).

**Figure 1:**
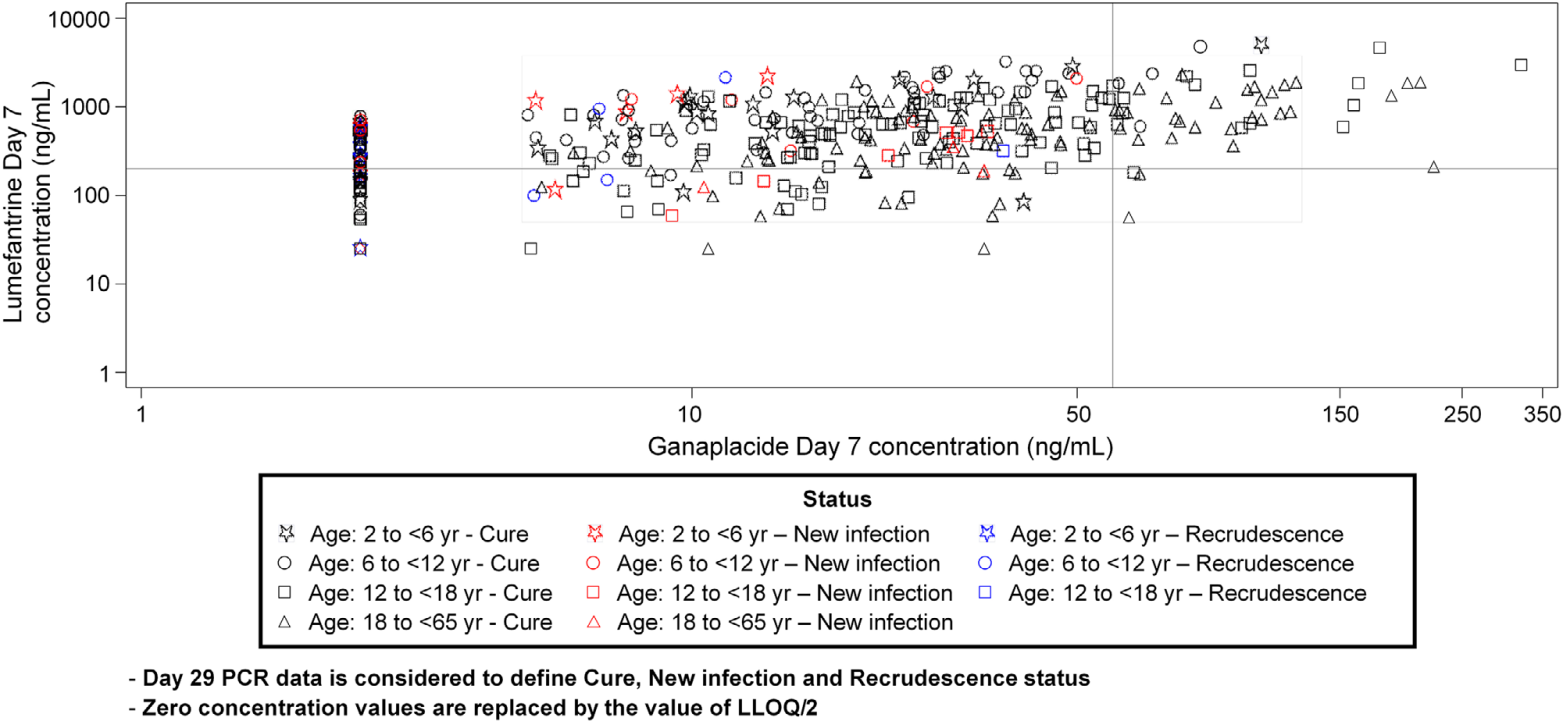
Scatter plot of ganaplacide and lumefantrine concentrations at Day 7 (C_168 h_) for all study parts, PK analysis set.

Figure 1 shows treatment outcomes at Day 29 by ganaplacide and lumefantrine exposure (C_168 h_); higher exposures appear to correlate with better efficacy, although ganaplacide C_168 h_ is unlikely to be clinically relevant, given the role of this compound in the combination.

## Discussion

This is the largest study completed to date with the novel imidazolopiperazine antimalarial ganaplacide, and the first to investigate the combination of ganaplacide with lumefantrine, the latter as the novel solid dispersion formulation.^11^ The study demonstrated that ganaplacide plus lumefantrine was effective and well tolerated in the treatment of uncomplicated falciparum malaria in adults, adolescents, and children over 2 years of age^2^ and confirmed the dose selection for a currently ongoing Phase III study (NCT05842954). The study provided for the first time assessment of the pharmacokinetics of ganaplacide in children, and in combination with lumefantrine. The study also represents the largest population in which ganaplacide pharmacokinetics have been assessed. The pharmacokinetic analyses in this study were prospectively defined in the study protocol.

In the pharmacokinetic run‐in part of this study, co‐administration of ganaplacide with lumefantrine under fasted conditions did not result in clinically relevant increases in ganaplacide or lumefantrine exposure, and no adjustments to ganaplacide or lumefantrine doses were considered necessary. Ganaplacide was rapidly absorbed with a mean t_max_ of approximately 4 h. Data from the run‐in part and Parts A and B of the study indicated that ganaplacide exposure was dose proportional, and was generally similar between adults and children. C_max_ and AUC_0‐24 h_ for ganaplacide were very similar across different age groups. It should be noted that the sample size in the lowest body weight group (10 to <15 kg) is small and the pharmacokinetic data from this population should be interpreted with caution. A currently ongoing Phase II study (NCT04546633) that also includes malaria patients in the ≥6 months to <2 years age group, and the ongoing Phase III study (NCT05842954) will provide further information on pharmacokinetics in very young children.

Previous data on ganaplacide pharmacokinetics in patients with malaria were reported from a trial performed in Thailand and Vietnam.^4^ Adult patients with *P. falciparum* or *P. vivax* malaria were treated with single 800 mg ganaplacide doses or 400 mg given once daily for 3 days. Ganaplacide was administered as monotherapy. Ganaplacide t_max_ was 3 h, with C_max_ after 400 mg doses of 795 ng/mL in *P. vivax* patients and 856 ng/mL in patients with *P. falciparum* malaria. AUC_0‐24 h_ was 9470 and 10,100 ng•h/mL, respectively. These data are comparable to those obtained with 400 mg ganaplacide doses in the current study. Pharmacokinetic data for 800 mg doses of ganaplacide in the study by White et al^4^ were also consistent with those in the current study.

Data from the current study (and particularly those based on sparse sampling) for the 400 and 800 mg ganaplacide doses were also broadly consistent with those obtained following administration of the same dose levels (single doses) to healthy adult volunteers.^9^, ^13^ Pharmacokinetic data for ganaplacide were also provided in an experimental *P. falciparum* infection study of antimalarial prophylaxis in healthy volunteers,^7^ where doses of ganaplacide used included 800 mg, and the exposure in terms of C_max_ and AUC_0‐24 h_ was comparable to that in the current study.

Lumefantrine pharmacokinetics after administration of the solid dispersion formulation has been assessed previously in healthy volunteers.^11^ Among the doses tested in that study was 960 mg, as used in a range of regimens in Parts A and B of the current study. As the participants in the previous study^11^ were all adult males, data from Part A of the current study provide a more meaningful basis for comparison to the data in healthy volunteers. The pharmacokinetics of lumefantrine in the current study are consistent with those in healthy volunteers receiving the same dose of LUM‐SDF, with similar t_max_, and comparable exposure in terms of C_max_ and AUC_0‐24 h_. Lumefantrine exposure was under dose‐proportional, and as expected, showed high interindividual variability, but was comparable across age and body weight groups.

The concentration of antimalarials 7 days or 168 h after dosing has been suggested as a predictor of efficacy.^14^, ^15^ With combination therapy, C_168 h_ is more clinically relevant for the longer‐acting partner. In this combination, ganaplacide is the fast‐acting partner used to achieve rapid initial parasite clearance (with a half‐life of approximately 24 to 33 h) and as such ganaplacide C_168 h_ is unlikely to be clinically relevant. More important parameters for ganaplacide are C_max_ and AUC_0‐24 h_, which are consistent across age and body weight groups.

In this combination, lumefantrine (half‐life 31 to 81 h in this study) is the long‐acting partner to maintain efficacy and prevent recrudescence. Lumefantrine exposure in terms of C_168 h_ was generally numerically higher with the 3‐day ganaplacide/LUM‐SDF regimen than with artemether‐lumefantrine administered as per current recommendations. Assessment of the relationship between exposure and the occurrence of recrudescence in this study suggested that lower lumefantrine C_168h_ was more likely to be associated with treatment failure, although this has not yet been formally analyzed. In the previous study of prophylaxis, Kublin et al^7^ also assessed the exposure‐response relationship for ganaplacide, observing a relationship between increased drug exposure and prophylactic efficacy; ganaplacide concentration 24 h after dosing was identified as the best predictor of response, with a concentration of 21.5 ng/mL ensuring a 95% chance of preventing infection. White et al,^4^ in their study in patients infected with *P. vivax* or *P. falciparum*, did not observe a clear relationship between ganaplacide exposure and initial parasitological response (in terms of parasite clearance time or parasite clearance half‐life), but did note that patients with *P. falciparum* recrudescence were more likely to have lower plasma concentrations (less than 58 ng/mL, more than double the 99% in vitro inhibitory concentration) than those without recrudescence. However, this is probably less relevant when ganaplacide is used in combination with lumefantrine than as monotherapy.

In conclusion, in this study of the novel antimalarial ganaplacide in combination with LUM‐SDF, the pharmacokinetic data indicate that the exposures of ganaplacide and lumefantrine needed for efficacy were obtained using the dose regimen 400 mg ganaplacide plus lumefantrine 960 mg once daily for 3 days under fasted conditions, in adults, adolescents, and children. A second Phase II study (NCT04546633) and a pivotal Phase III study (NCT05842954) will provide further data for this novel antimalarial combination.

## Conflicts of Interest

Ramachandra Sangana, Celine Risterucci, Guoqin Su, Rashidkhan Pathan, Havana Chikoto, Katalin Csermak, Cornelis Winnips, Jie Zhang, and Julia Zack are employees and stockholders of Novartis. Myriam El Gaaloul is an employee of Medicines for Malaria Venture. Anne Claire Marrast is an employee of Medicines for Malaria Venture and a stockholder of Novartis, Alcon, and Idorsia. The other authors declare no conflicts of interest.

## Funding

The study was funded by Novartis and Medicines for Malaria Venture (in collaboration with the Bill & Melinda Gates Foundation).

## Supporting information



Supporting Information

## Data Availability

Individual participant data will be shared with qualified external researchers under a signed data sharing agreement. All data are anonymized in line with applicable laws and regulations and available from the time of publication. The study protocol and statistical analysis plan are available on ClinicalTrials.gov (NCT03167242). Requests for data are reviewed and approved by an independent review panel on the basis of scientific merit. Data are available at https://www.clinicalstudydatarequest.com/
