# Supplementary material for: Pharmacokinetics of Ganaplacide and Lumefantrine in Adults, Adolescents, and Children with Plasmodium falciparum Malaria Treated with Ganaplacide Plus Lumefantrine Solid Dispersion Formulation: Analysis of Data from a Multinational Phase 2 Study
Source: J Clin Pharmacol. 2024 Sep 29;65(2):179–89. doi: 10.1002/jcph.6138 (PMC11771541; doi:10.1002/jcph.6138)

Ramachandra Sangana, Bernhards Ogutu, Adoke Yeka, Sylvia Kusemererwa, Halidou Tinto, Andre Offianan Toure, Afizi Kibuuka, Moussa Lingani, Carlos Lourenço, Ghyslain Mombo-Ngoma, Videlis Nduba, Tiacoh Landry N'Guessan, Guétawendé Job Wilfried Nassa, Mary Nyantaro, Lucas Otieno Tina, Anup Anvikar, Abhinav Sinha, Grace Kaguthi, Bakary Fofana, Martin Peter Grobusch, Myriam El Gaaloul, Anne Claire Marrast, Rashidkhan Pathan, Havana Chikoto, Katalin Csermak, Celine Risterucci, Guoqin Su, Cornelis Winnips, Jie Zhang, and Julia Zack

Pharmacokinetics of ganaplacide and lumefantrine in adults, adolescents, and children with Plasmodium falciparum malaria treated with ganaplacide plus lumefantrine solid dispersion formulation: analysis of data from a multinational Phase 2 study

Online Supplementary Material

Table S1 Blood sampling schedules for pharmacokinetic analysis

Study day	Time post-dose (hours)	1-day regimens	2-day regimens	3-day regimens	Artemether-lumefantrine
Rich PK sampling (PK run-in part and part A)					
1	0	•	•	•	
	1	•	•	•	
	3	•	•	•	
	6	•	•	•	
	12	•	•	•	
	18	•			
2	24	•	•	•	
	27		•		
	30	•	•		
	36		•		
3	48	•	•	•	
	51			•	
	54			•	
4	72		•	•	
5	96	•	•	•	
8	168	•	•	•	
15	336			•	
Sparse PK sampling (Part A)					
1	3	•	•	•	
	6	•	•	•	•
	18	•			
2	24	•	•	•	•
	27		•		
	30	•	•		
3	48		•	•	•
	51			•	
	54			•	
	68				•
4	72			•	
8	168	•	•	•	•
Sparse PK sampling (Part B)					
1	3	•			
	6	•			
2	24	•	•	•	•
	27		•		
	30		•		
3	48		•	•	•
	51			•	
	54			•	
	68				•
4	72			•	
8	168	•	•	•	•

Table S2 Prohibited concomitant medications

CYP enzymes/ transporter	Sensitive substrates[a]	Substrates with narrow therapeutic range[b]
CYP2D6	Atomoxetine, desipramine, dextromethorphan, metoprolol, nebivolol, perphenazine, tolterodine, venlafaxine	Thioridazine, neuroleptics, flecainide, metoprolol, and tricyclic antidepressants such as imipramine, amitriptyline, clomipramine
CYP3A	Alfentanil, aprepitant, budesonide, buspirone,conivaptan, darifenacin, darunavir, dasatinib, dronedarone, eletriptan, eplerenone, everolimus, felodipine, indinavir, fluticasone, lopinavir, lovastatin, lurasidone, maraviroc, midazolam, nisoldipine, quetiapine, saquinavir, sildenafil, simvastatin, sirolimus, tolvaptan, tipranavir, triazolam, vardenafil	Alfentanil, astemizole, cisapride, cyclosporine, dihydroergotamine, ergotamine, fentanyl, pimozide, quinidine, sirolimus, tacrolimus, terfenadine
CYP2B6[c]	Bupropion, efavirenz	
CYP2C8	Repaglinide[d]	Paclitaxel
CYP2C9	Celecoxib	Warfarin, phenytoin
OATP1B1	Bosentan, pravastatin	

[a]Sensitive CYP substrates refers to drugs whose plasma AUC values have been shown to increase 5-fold or higher when co-administered with a known CYP inhibitor.

[b]CYP substrates with narrow therapeutic range refers to drugs whose exposure-response relationship indicates that small increases in their exposure levels by the concomitant use of CYP inhibitors may lead to serious safety concerns (e.g., Torsades de Pointes).

[c]The AUC of these substrates were not increased by 5-fold or more with a CYP2B6 inhibitor, but they represent the most sensitive substrates studied with available inhibitors evaluated to date.

[d]Repaglinide is also a substrate for OATP1B1, and KAF156 potentially inhibits therapeutic OATP1B1 as well.

Figure S1 Box and whisker plots for $AUC_{0\text{-}24h}$ from Day2/Day 3 for ganaplacide and lumefantrine, by PK sampling type and treatment group, PK run-in cohort and Part A, PK analysis set

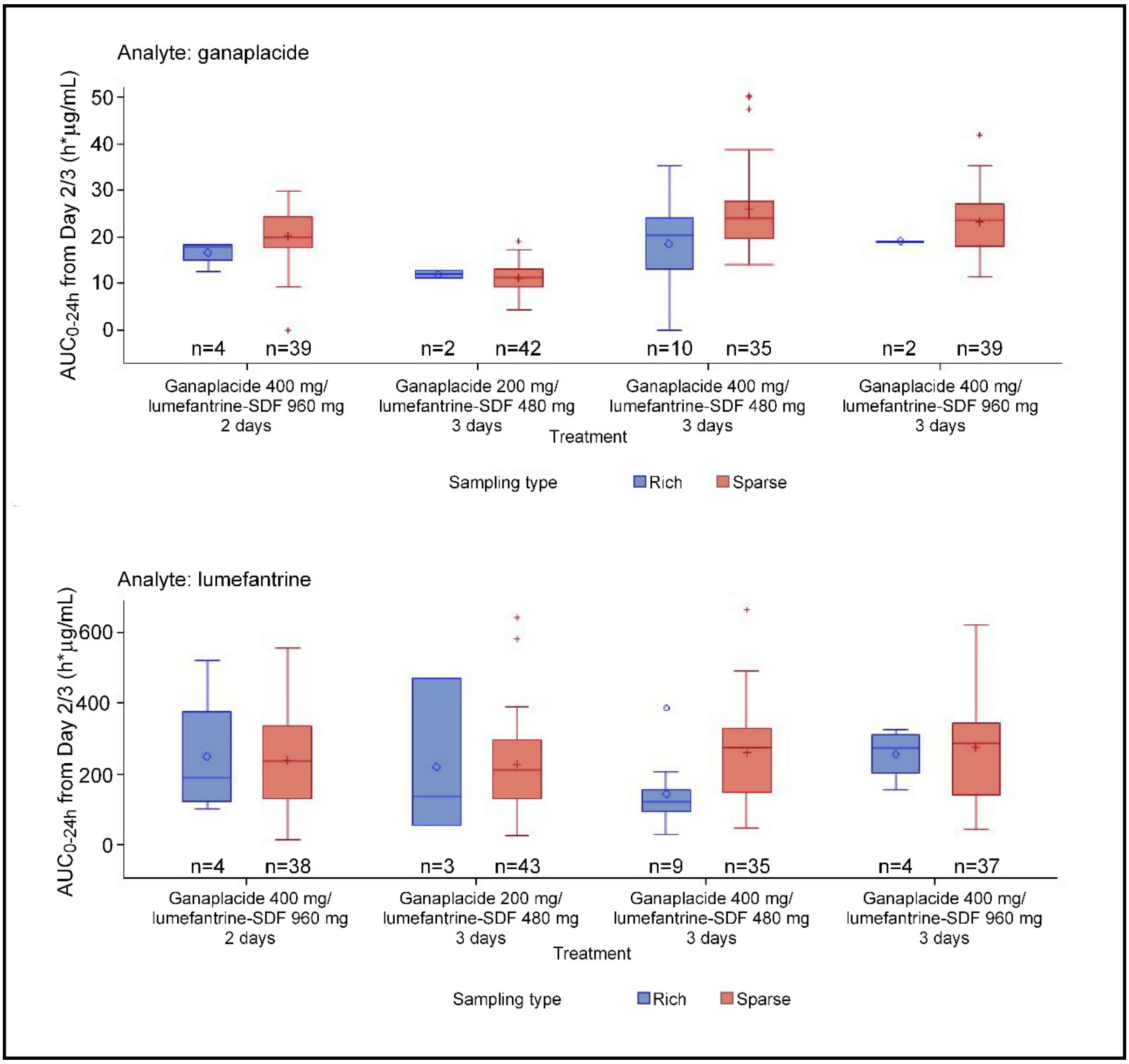

Supplement: Supplementary file 1 — Supporting Information [file JCPH-65-179-s001.pdf]
